# Angiopoietin-2 promotes ER+ breast cancer cell survival in bone marrow niche

**DOI:** 10.1530/ERC-16-0086

**Published:** 2016-08-01

**Authors:** Hyun Ho Han, Baek Gil Kim, Joo Hyun Lee, Suki Kang, Ji Eun Kim, Nam Hoon Cho

**Affiliations:** 1 Brain Korea 21 Plus Project for Medical ScienceYonsei University College of Medicine, Seoul, South Korea; 2 Department of PathologyYonsei University College of Medicine, Seoul, South Korea; 3 Severance Biomedical Science Institute (SBSI)Yonsei University College of Medicine, Seoul, South Korea; 4 Global 5-5-10 System BiologyYonsei University, Seoul, South Korea

**Keywords:** breast, bone, metastasis, endocrine therapy resistance, cell signaling

## Abstract

In estrogen receptor-positive (ER+) breast cancer, it is recognized that metastases may develop after a long period of dormancy. Bone marrow (BM) vascular niche is where the dormant tumor cells are most likely to reside. So far, it is not fully understood why the dormant tumor cells become proliferative and eventually generate tumor. We hypothesized that therapeutic or menopause-related estrogen depletion may be the switch behind dormant ER+ tumor cell awakening in BM. We utilized an existing experimental model of BM endothelial niche that can simulate ER+ tumor cell dormancy to test our hypothesis. In results, estrogen depletion paradoxically promoted ER+ tumor cell proliferation in the BM endothelial niche, and their molecular phenotype shifted from dormant to awaken. Following estrogen depletion, the BM niche cells produced angiopoietin-2 (*ANGPT2*), which destabilized niche endothelium by interfering *ANGPT1*/Tie2 signaling, and promoted ER+ tumor cell survival under estrogen deficiency via cell surface integrin &1. Knockdown of *ANGPT2* completely negated ER+ tumor cell awakening in the niche. Furthermore, ANGPT2 expression in ER+ tumor human samples was associated with increased risk of distant metastasis only in those who underwent adjuvant estrogen depletion therapy, not in those who did not undergo adjuvant therapy. In conclusion, we demonstrate that *ANGPT2* signaling activated after estrogen depletion paradoxically triggers ER+ tumor cell awakening from dormancy in their BM niche, partly indirectly via endothelial Tie2 receptor and partly directly via tumor cell surface integrin &1.

## Introduction

Tumor cells frequently escape the primary site and disseminate into multiple distant organs such as bone marrow (BM) (Aguirre-Ghiso 2007). The cells may remain there in a dormant, growth-arrested state for a long time. Disseminated dormant tumor cells can threaten patients’ survival, since the tumor cells are resistant to standard adjuvant therapies, can evade current monitoring protocols (Braun *et al*. 2000) and eventually be reactivated to form metastatic tumors (Janni *et al*. 2005, Janni *et al*. 2011). Recurrence after long period of dormancy is especially common in estrogen receptor-positive (ER+) breast cancer (Han *et al*. 2016, Zhang *et al*. 2013). While estrogen receptor-negative (ER&) cancer rarely recurs after 5 years, ER+ cancer often recurs even decades after otherwise successful primary tumor treatment. Understanding the molecular mechanisms of tumor dormancy and reactivation can facilitate the development of therapeutic strategies against metastatic recurrence (Giancotti 2013), comprising most of the ER+ breast cancer patient mortality (Goss & Chambers 2010).

Tumor cell dormancy seems to be regulated by a special environment, called a niche, which exists in lymph node, lung or BM (Aguirre-Ghiso 2007). Since niche exists only sparsely and dormant tumor cells are even sparser, human or animal study of tumor cell dormancy in niche is a technical challenge itself. *In vitro* models of tumor dormancy, therefore, have crucial importance in this field (Barkan & Green 2011). For instance, the BM niche contains many types of stromal cells, including mesenchymal stem cells (MSCs), osteoblasts, pericytes, fibroblasts and endothelial cells (ECs) (Mendez-Ferrer *et al*. 2010, Kunisaki *et al*. 2013). Among the cellular components, the role of EC is an emerging topic of importance (Ghajar 2015). Ghajar and coworkers (2013) found out that endothelial components regulate breast tumor cell dormancy in lung and BM perivascular niches by utilizing both *in vitro* co-culture models of BM niche and *in vivo* models. Similarly, Marlow and coworkers (2013) developed a three-dimensional co-culture model of BM niche by mixing MSCs, osteoblasts and ECs, which successfully reproduced dormancy of bone metastatic breast cancer in human.

In general, ER+ tumor cells need estrogen for survival and proliferation. However, many metastatic events of ER+ breast cancer come after years of adjuvant antiestrogen therapy or after menopause when systemic estrogen levels become extremely low (Zhang *et al*. 2013). Furthermore, it is argued that estrogen depletion may promote, paradoxically, breast cancer metastasis. For example, Ottewell and coworkers (2014) reported that ER- breast cancer bone metastasis in animal model did not decrease but increased after ovariectomy, especially via osteoclast activation. Similarly, McBryan and coworkers (2015) reported that antiestrogen treatment using tamoxifen promoted particularly metastatic recurrence of endocrine-resistant ER+ breast cancer. Both results imply that estrogen deficiency can remodel the microenvironment of secondary organs, which may also support survival and metastasis of endocrine-sensitive ER+ tumor cells.

Angiopoietin-2 (*ANGPT2*) may be the switch underling ER+ breast cancer relapse after estrogen depletion. *ANGPT2* is expressed by endothelium and acts as an autocrine or paracrine antagonist of *ANGPT1* (Augustin *et al*. 2009). It has been gaining attention as a promising target in cancer therapy, and specific inhibitors are now available (Saharinen *et al*. 2011, Eroglu *et al*. 2013). In primary organ, *ANGPT2* directly stimulates tumor angiogenesis (Eroglu *et al*. 2013). In metastatic sites, *ANGPT2* loosens the endothelial cell–cell junction, which enhances extravasation of disseminated tumor cells (Schulz *et al*. 2011, Holopainen *et al*. 2012, Avraham *et al*. 2014). Yet, its role after tumor cell extravasation, especially on tumor cell dormancy and reawakening, has not yet been explored. Since estrogen regulates *ANGPT1, 2* expressions in other tissues (Ardelt *et al*. 2005, Matsuoka-Sakata *et al*. 2006, Bonagura *et al*. 2010, Harfouche *et al*. 2011), we hypothesized that estrogen deficiency may modulate *ANGPT2* signaling in the BM niche, triggering ER+ tumor cell awakening from dormancy. Herein, we demonstrate that estrogen-deficient BM niche overexpresses angiopoietin-2, which negates ER+ tumor cell dormancy and eventually promotes estrogen-independent tumor growth.

## Materials and methods

### Cell lines and culture conditions

Breast cancer cell lines MCF7, BT474, MDA-MB-361 and MDA-MB-231 were obtained from the American Tissue Culture Collection (ATCC) and grown in complete RPMI-1640 medium (Gibco) supplemented with 10% fetal bovine serum (FBS; Gibco), 100 unit/mL penicillin and 100 &g/mL streptomycin (passage number ranged from 9 to 15). Above cell lines were authenticated by standard short tandem repeat (STR) DNA typing methodology before being purchased from the ATCC. Primary human umbilical vein endothelial cells (ECs) at second passage were obtained commercially (C-12203, lot #3070401, PromoCell GmbH, Heidelberg, Germany) and grown in endothelial cell growth medium 2 (EGM2; PromoCell, Heidelberg, Germany) in a humidified chamber (37°C, 5% CO_2_). Primary human bone marrow mesenchymal stem cells (BM MSCs) at second passage were obtained from Yonsei Cell Therapy Center (lot #B090429-04; #B110124-07, Seoul, Korea). BM MSCs were maintained in low glucose Dulbecco’s Modified Eagle Medium (DMEM; Gibco) supplemented with 10% FBS, 100 unit/mL penicillin and 100 &g/mL streptomycin. Endothelial cells (ECs) and BM MSCs isolated between passages 5 and 10 were used in these experiments.

### Generation of tumor cells expressing fluorescent tags

Tumor cell lines were tagged with red fluorescent protein (RFP) or enhanced green fluorescent protein (GFP) using a lentiviral transduction system. Briefly, pLenti CMV/TO Puro empty vector was obtained from Addgene (Addgene plasmid 17482; Cambridge, MA, USA) 49. RFP and GFP (sequences obtained from GenBank) were cloned into pLenti CMV/TO Puro empty vector. Lentivirus was generated by co-transfection of packaging vectors pMDLg/pRRE, pMD2G, pRSV-Rev (Addgene plasmids 12251, 12253, and 12259) and pLenti CMV/TO Puro-RFP or pLenti CMV/TO Puro-GFP into 293T cells with 2.5 M calcium chloride. RFP- or GFP-expressing tumor cells lines were generated by lentiviral infection and selection for 1 week in 1 &g/mL puromycin.

### *In vitro* model of bone marrow niche

MSCs and ECs were co-cultured in EGM2 for 5–7 days to reach confluence. For three-dimensional (3D) culture, growth-factor-reduced, phenol-red-free Matrigel matrix (Corning) was used to coat the vessels before cell seeding. To discriminate ECs from MSCs, ECs were stained with carboxyfluorescein succinimidyl ester (CFSE, Life Technologies) before co-culture. Cell numbers and culture volumes were as follows: 5 × 10^3^ MSCs, 2 × 10^3^ ECs in microfluidic plates. 5 × 10^4^ MSCs, 2 × 10^4^ ECs and 200 &L EGM2 per well in 96-well microplates; 2 × 10^5^ MSCs, 5 × 10^4^ ECs and 2 mL EGM2 per well in 6-well microplates; and 2 × 10^6^ MSCs, 5 × 10^5^ ECs and 10 mL EGM2 in 100 mm dishes.

### Monitoring tumor cell proliferation using continuous fluidic cell culture system

A microfluidic live-cell imaging platform (CellASIC ONIX Microfluidic Platform, EMD Millipore) was utilized to supply nutrients and oxygen and remove wastes with minimal stress to cells. Cell culture was performed according to the manufacturer’s instructions. Briefly, 10 &L MSC and EC cell suspensions (1 × 10^6^ total cells/mL) was loaded onto a microfluidic culture plate (M04S, EMD Millipore), which has a compatible culture volume as that of 384-well microplate. The plate was attached to the platform controlling perfusion flow, temperature and gas composition. An inverted fluorescence microscope was used for live-cell imaging with the 40× objective. The cells were incubated (EGM2, 37°C, 5% CO_2_) for 5–7 days until reaching confluence. Then, fluorescence-expressing tumor cells were seeded sparsely onto BM niche culture plates. Tumor cell seeding numbers were as follows: 0.5 × 10^2^ cells per well in microfluidic plate, 2 × 10^2^ cells per well in 96-well microplates, 4 × 10^3^ cells per well in 6-well microplates and 2 × 10^4^ MCF7 in 100 mm dishes. For microfluidic culture, tumor cell proliferation was monitored by capturing time-lapse images of the cells using a fluorescence microscope (Nikon Eclipse Ti, Nikon Instruments). For conventional culture, a fluorescence microplate reader (Varioskan Flash Multimode Reader, Thermo Scientific) was used to measure cell fluorescence every 24 h after cell seeding. Fluorescence intensity was measured using bottom optic readings. Excitation/emission wavelengths were 553/574 nm for RFP and 488/507 nm for GFP.

To compare proliferative capacities of tumor cells co-cultured with different stromal cell compositions (MSCs, MSCs-ECs or no stromal cells as a control), all cells were harvested by trypsinization after co-culturing for up to 7 days. Fluorescence-positive cells were sorted and collected using fluorescence-activated cell sorting (FACS) (FACSAria cell sorter; BD Bioscience, San Jose, CA, USA), and seeded onto new empty plates (2 × 10^3^ cells per well in 96-well microplates). Cell proliferative capacity was assessed again by measuring fluorescence intensity every 24 h.

### Flow cytometry to detect ERK1/2 and p38 activities

GFP-expressing tumor cells were cultured alone or with MSCs and/or ECs in 100 mm dishes for 7 days. Cells were trypsinized, washed with phosphate-buffered saline (PBS) and fixed with 4% paraformaldehyde for 10 min. The fixed cells were chilled on ice and permeabilized with 90% methanol for 30 min. Then, 1 × 10^6^ cells per experimental condition were aliquoted, washed and resuspended in 100 &L fluorochrome-conjugated primary antibodies against p38, phosphorylated p38 (Santa Cruz Biotechnology), ERK1/2 and phosphorylated ERK1/2 (Cell Signaling Technology) at the manufacturer’s recommended concentrations, and incubated for 1 h. For isotype control, fluorochrome-conjugated rabbit IgG was used at the same concentration. Cells were washed, resuspended in PBS, sorted for GFP fluorescence and analyzed using the FACSAria cell sorter.

### Estrogen depletion and supplement in culture

FBS stripped with charcoal-dextran was purchased from Gemini Bio-Products (Gemini Bio-Products, West Sacramento, CA, USA). To evaluate the effect of estrogen depletion on BM niche, MSCs and/or ECs were cultured in phenol-red-free RPMI 1640 containing 10% charcoal-stripped FBS supplemented with or without 200 pg/mL 17&-estradiol (E2758, Sigma-Aldrich). Because the charcoal-stripping process eliminates estrogen and other steroidal hormones essential for primary EC survival, hydrocortisone (0.2 &g/mL) was added to the final medium. To supplement estrogen, 17-& estradiol (Sigma-Aldrich) was added.

### Modulation of receptor–ligand interaction

To evaluate the effect of angiopietin-1, 2 on tumor cell proliferation, cultures were treated with 0, 50, 100 or 500 ng/mL recombinant human angiopoietin-1 and recombinant human angiopoeitin-2 (R&D Systems). To block angiopoietin-1 and Tie2 receptor interaction, cultures were treated with 10 &g/mL of human Tie2 affinity-purified polyclonal antibody (R&D Systems). To knockdown angiopoietin-2 in culture, short-inhibiting RNAs (siRNAs) were used. Three different siRNAs that are known to block specifically angiopoietin-2 mRNA were generated:

    #1 5&-GGAAGAGCAUGGACAGCAUAGGA-3& (D’Souza *et al*. 2012)

    #2 5&-AGAACCAGACGGCUGUGAUGAUAGAAA-3& (Daly *et al*. 2006)

    #3 5&-CCAGACGGCUGUGAUGAUA-3& (Mangan *et al*. 2007)

Negative and positive control siRNAs were obtained from GenePharma (Suzhou, China). Cells were prepared and cultured as described above and siRNAs were transfected using transfection reagent G-Fectin (Genolution Pharmaceuticals. Seoul, Korea) according to the manufacturer’s protocol. Briefly, 1 &L G-Fectin and 5 pmol siRNA were incubated in 50 &L PBS at room temperature for 10 min, and added to cell culture plate (24-well). Culture medium was replaced 8 h after transfection to minimize cytotoxicity. Angiopoietin-2 knockdown was assessed by PCR using total RNA extracted from cells 48 h post-transfection.

To knockdown ITGB1, we used the SureSilencing shRNA plasmid for human ITGB1 (KH00650G for the GFP) and a scrambled sequence negative control plasmid (SABiosciences, Frederick, VA, USA), as described previously (Choi *et al*. 2012). The shRNA target sequence for ITGB1 was as follows: 5&-TGT GCT CAG TCT TAC TAA TAA-3&. The cells were seeded and transfected using the Attractene Transfection Reagent (Qiagen) according to the manufacturer’s protocol.

### RNA extraction and reverse transcription polymerase chain reaction (RT-PCR)

As described previously (Kang *et al*. 2013), total RNA was isolated from cells using an RNeasy Protect Mini Kit (Qiagen) according to the manufacturer’s protocol. The Super-Script III Reverse Transcriptase kit (Invitrogen) was used to synthesize cDNA. Polymerase chain reactions (PCRs) were performed with HotStarTaq DNA polymerase (Qiagen) and the following conditions: denaturation at 95°C for 15 min, and 28 cycles of 95°C for 40 s, 52°C for 1 min and 72°C for 1 min, with a final extension for 10 min at 72°C. Expression levels of GAPDH were assessed as an internal control in all reactions. The following primers were used for PCR: forward primer for angiopoietin-1 5&-GAAGGGAACCGAGCCTATTC-3&, reverse primer 5&-GGGCACATTTGCACATACAG-3&; forward primer for angiopoietin-2 5&-TGGGATTTGGTAACCCTTCA-3&, reverse primer 5&-GGTTGGCTGATGCTGCTTAT-3&.

### Western blotting assay

As described previously (Kang *et al*. 2013), cells were trypsinized and lysed by Pro-Prep protein extraction kit (iNtRON Biotechnology, Seongnam, Korea). Equal amounts of protein extracts (20 &g) were separated by sodium dodecyl sulfate–polyacrylamide gel electro­phoresis and transferred to a nitrocellulose membrane (Invitrogen). Blots were blocked with 5% nonfat dry milk at room temperature. The blots were incubated with antibodies specific for angiopoietin-1 (ab183701, Abcam), angiopoietin-2 (ab155106, Abcam), phosphorylated Tie2 (Tyr992, #4221, Cell Signaling Technology), Tie2 (sc-9026), thrombospondin-1 (sc-59886) and &-actin (Santa Cruz Biotechnology) at specific dilution, followed by incubation with peroxidase-labeled secondary antibodies. Immunoreactive proteins were visualized using an enhanced chemiluminescence detection kit (Santa Cruz Biotechnology). Blot images were captured using ImageQuant LAS 4000 biomolecular imager (GE Healthcare Life Sciences). Blot intensities were quantified using ImageJ 1.48v software (http://imagej.nih.gov/ij) (Schneider *et al*. 2012).

### Graphics and statistical analysis

For survival analysis of human patients, statistical significance was determined using Kaplan–Meier method and log-rank test. For *in vitro* experiments, statistical significance was determined using Student’s *t*-test and ANOVA. Results were considered to be significant at *P* < 0.05. Statistical analyses were computed with IBM SPSS Statistics 20 software. All graphical presentations were created using GraphPad Prism 6 software.

## Results

### Angiopoietin-1, 2 regulate ER+ tumor cell dormancy in BM endothelial niche

**Figure 1:**
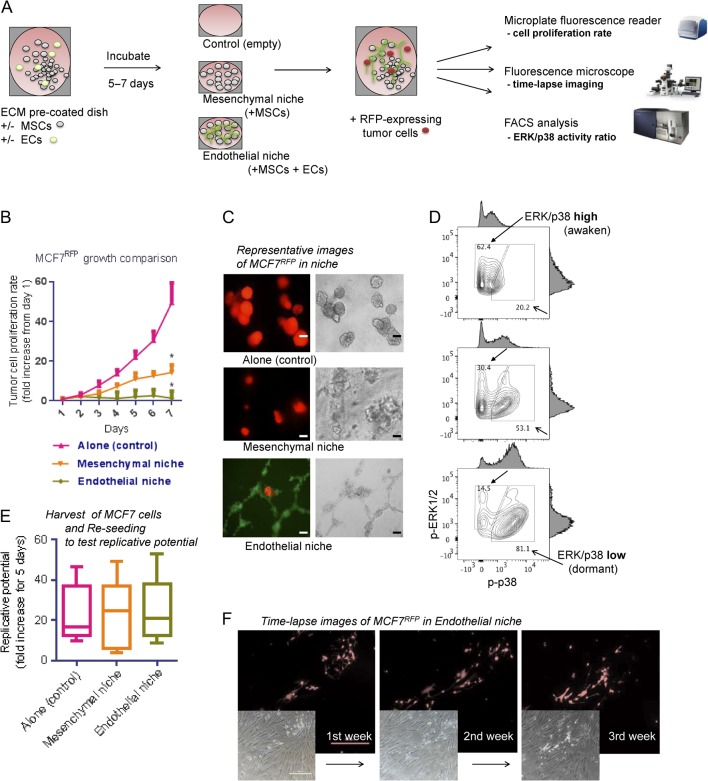
ER+ breast tumor cell MCF7 dormancy in bone marrow endothelial niche models. (A) Experimental scheme. BM MSCs and/or ECs were seeded on 96-well microplates and incubated until reaching confluence. For 3D cultures, Matrigel was coated (150 &L/cm^2^) onto the culture plate. Then, RFP-expressing tumor cells were sparsely seeded (200 cells/well) onto the niche cells or empty surfaces (control). Tumor cell proliferations were assessed by fluorescence intensity reader and microscope, and fluorescence-activated cell sorting (FACS). (B) Seven-day proliferation rates of MCF7 cells in niches (five sample sets per group; error bars: ± standard deviation (s.d.) *P < 0.05). (C) Representative images of tumor cells in the niches, captured after 7 days of co-culture (left: fluorescence (red and green); right: bright-field. scale bar = 100 &m). For visualization, ECs were prestained with CFSE (green). (D) FACS analysis of ERK1/2 and p38 MAR kinase activities of MCF7 tumor cells in niche. Upper: tumor cell alone; middle: tumor cell in mesenchymal niche; lower: tumor cell in endothelial niche. Blank arrows: ERK/p38 ratio-low (dormant) population; filled arrows: ERK/p38 ratio-high (awaken) population. (E) Replicative potentials of dormant MCF7 tumor cells. Tumor cells were harvested from niche and replated in 96-well microplates (500 cells/well). Box-plots represent fluorescence intensity fold-changes for 7 days (five samples per condition. horizontal bar = mean). (F) Time-lapse images of tumor cell dormancy in niche. MCF7^RFP^ cells were cultured in endothelial niche for up to 21 days (fresh medium replaced every 48 h). Cells remain at almost the same number during the period. (Upper: fluorescence (red); lower: bright-field. scale bar = 500 &m). BM MSC, bone marrow-derived mesenchymal stem cell; EC, endothelial cell; ER, estrogen receptor; MAP, Mitogen-activated protein; p-ERK1/2, phosphorylated ERK1/2; p-p38, phosphorylated p38; RFP, red fluorescence protein. A full colour version of this figure is available at http://dx.doi.org/10.1530/ERC-16-0086.

Angiopoietin-1/Tie2 signaling is active in the BM niche, mainly participating in hematopoietic stem cell quiescence (Arai *et al*. 2004). If angiopoietin signaling does have a role in ER+ tumor metastatic recurrence, it is probably working also in the BM niche where disseminated tumor cells are most likely to reside. To test the impact of angiopoietin signaling on ER+ breast tumor dormancy in BM niche, we utilized existing *in vitro* models (Barkan & Green 2011, Ghajar *et al*. 2013, Marlow *et al*. 2013). Briefly, to create a microenvironment similar to BM niche, human BM-derived MSCs and/or ECs were co-seeded onto extracellular matrix-coated microplates (Fig. 1A). These were designated as ‘mesenchymal niche’ or ‘endothelial niche’, based on the presence of ECs. Without ECs, BM-derived stromal cells (mesenchymal niche) generally support breast tumor cell growth, whereas EC-contained niche (endothelial niche) inhibits tumor cell growth (Marlow *et al*. 2013). To optimize the system for our studies, a few modifications have been introduced: a commercial continuous fluidic cell culture system was used to supply nutrients and oxygen and remove wastes with minimal stress to cells during long-term culture; charcoal-stripping fetal bovine serum (FBS) was used to eliminate serum-derived estrogen.

The niches were incubated until their cells reaching confluence in medium containing 10% charcoal-stripped FBS supplemented with estradiol (E_2_) 200 pg/mL. Then, fluorescence-expressing ER+ human breast tumor cells were seeded sparsely onto the niche surface. Stromal cells and tumor cell seeding densities were determined by references and our own preliminary studies (Supplementary Fig. 1, see section on supplementary data given at the end of this article). Tumor cell proliferation was monitored by its fluorescence visualization using a microplate reader and a microscope. MCF7 breast tumor cells exhibited significant growth suppressions in endothelial niche compared with those of the mesenchymal niche or cultured alone (Fig. 1B and C). The results were repeated in other ER+ tumor cell lines BT474 and MDA-MB-361 (Supplementary Fig. 2A and B). An ER-negative cell line MDA-MB-231 did not show growth suppression in endothelial niche (compared with those cultured alone), but its proliferation rate in mesenchymal niche was significantly higher than those in endothelial niche or cultured alone (Supplementary Fig. 2C). Mitogen-activated protein (MAP) kinases activity ratio of ERK1/2 and p38 (ERK/p38 ratio) can be used as an indicator of tumor cell dormancy and awakening: proliferative tumor cells exhibit high ERK/p38 ratio, while dormant tumor cells exhibit low ERK/p38 ratio (Sosa *et al*. 2011). Since both proteins are active in phosphorylated forms, we analyzed their phosphorylation status in MCF7 cells grown in mesenchymal niche and endothelial niche by FACS. Compared with those cultured alone or in mesenchymal niche, tumor cells in endothelial niche exhibited a shift toward low ERK/p38 ratio (Fig. 1D). When the tumor cells cultured in endothelial niche were harvested and reseeded in empty plates, they started replication and generated tumor, implying that their tumorigenic potentials were maintained (Fig. 1E). Growth suppression of tumor cells in our endothelial niche model could be extended up to 3 weeks (Fig. 1F).

**Figure 2:**
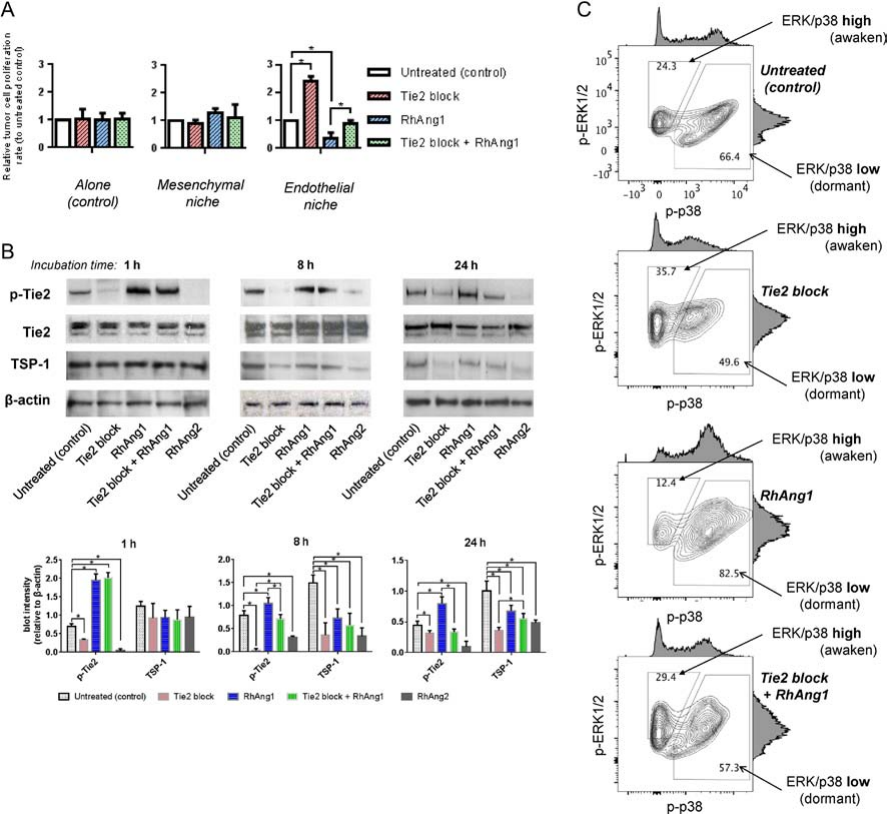
Disruption of endothelial angiopoietin-1/Tie2 signaling elicits awakening of dormant MCF7 tumor cells in endothelial niche. (A, B and C) To modulate endothelial angiopoietin-1/Tie2 signaling in culture, 500 ng/mL recombinant human angiopoietin-1 (RhAng1) or 10 &g/mL human Tie2 receptor-specific blocking antibody were treated on the niche models. ER+ tumor cell dormancy/awakening states in the niches were assessed by fluorescence intensity reader and FACS. (A) Relative tumor cell proliferation rates in niches. Cell proliferation rates for 3 days of RhAng1 and/or Tie2 blockade were analyzed. Data are presented as mean signaling intensity changes, relative to untreated control (*n* = 3 per condition. Error bar: ±s.d. *P < 0.05). (B) Endothelial niche Tie2 receptor phosphorylation and thrombospondin-1 (TSP-1) level changes upon Tie2 block, RhAng1, Tie2 block+RhAng1 or RhAng2 500 ng/mL. Above: representative lot images. Below: Blot quantification. Normalized to &-actin. Error bar: s.d. Cells were harvested after 1, 8 or 24 h of incubation. Experiments were done triplicated. (C) ERK1/2 and p38 MAR kinase activities of tumor cells in RhAng1 and/or Tie2 blocking antibody-treated niches. Filled arrows: ERK/p38 ratio-high population. Blank arrows: ERK/p38 ratio-low population. Black = untreated; red = Tie2 blocked; blue = RhAng1-treated; green = Tie2 blocked & RhAng1-treated endothelial niches. A full colour version of this figure is available at http://dx.doi.org/10.1530/ERC-16-0086.

**Figure 3:**
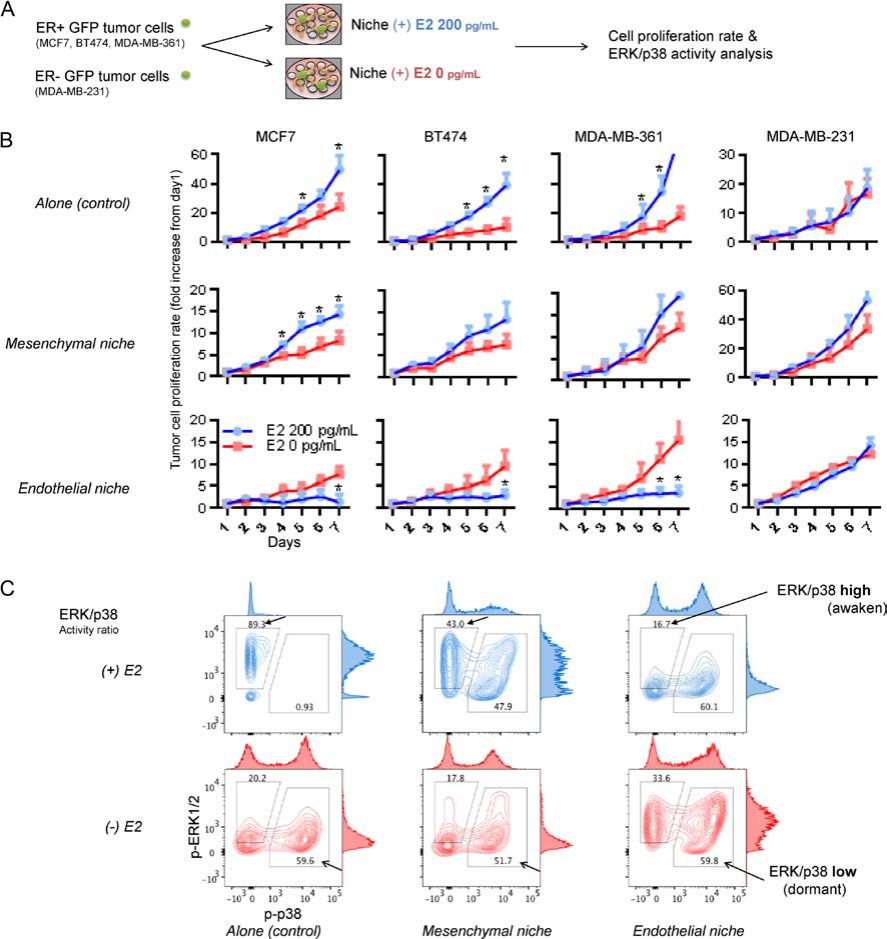
Increased ER+ tumor cell proliferation after estrogen depletion in bone marrow endothelial niche. (A) Experimental scheme. BM niche models were generated by co-culturing BM MSCs and/or ECs on ECM-coated microplates. GFP-expressing tumor cells were sparsely seeded (200 cells/well) onto the niche cells or empty surfaces (control). Tumor cell proliferations were compared in niches cultured using estrogen-supplemented (200 pg/mL) or un-supplemented (depleted) media. (B) Seven-day proliferation rates of tumor cells in niches. From left to right: ER+ MCF7, ER+ BT474, ER+ MDA-MB-361 and ER- MDA-MB-231. (Five sample sets per group; error bars: ±s.d. *P < 0.05). (C) FACS analysis of ERK1/2 and p38 MAR kinase activities of MCF7 tumor cells in estrogen-supplemented (blue) and estrogen-depleted (red) niches. Filled arrows: ERK/p38 ratio-high population. Blank arrows: ERK/p38 ratio-low population. A full colour version of this figure is available at http://dx.doi.org/10.1530/ERC-16-0086.

Vascular phenotype inside tumor can be switched by modulating angiopoeitin-1/Tie2 signaling (Reiss *et al*. 2009). We tested the effect of angiopoietin-1/Tie2 signaling modulation on tumor cell dormancy in BM endothelial niche. We added human Tie2-receptor-specific blocking antibody (10 &g/mL) to culture medium of endothelial niche. Compared with untreated control, the growth of ER+ tumor cells in endothelial niche significantly increased upon Tie2 blockade (Fig. 2A). In contrast, recombinant human angiopoietin-1 (RhAng1) 500 ng/mL treatment further suppressed tumor cell proliferation in endothelial niche (Fig. 2A). Again, Tie2 blockade significantly increased tumor cell proliferation in RhAng1-treated endothelial niche. Meanwhile, the growth of tumor cells in mesenchymal niche or those cultured alone were not affected by either Tie2 blockade or RhAng1 (Fig. 2A). Tumor cell proliferation rates in the endothelial niches were correlated with their Tie2 receptor activities and thrombospondin-1 (TSP-1) levels (Fig. 2B). Next, tumor cell’s ERK/p38 activity ratio was examined. Upon Tie2 blockade, ERK/p38 high (awaken) MCF7 cell population in endothelial niche increased while ERK/p38 low (dormant) population decreased (Fig. 3B). In contrast, RhAng1 further increased dormant tumor cell population in endothelial niche (Fig. 2B). Combination of RhAng1 treatment and Tie2 blockade recovered the activity ratio of ERK/p38 MAP kinases in similar degree to those of untreated control (Fig. 2B). We also tested the effect of recombinant human angiopoietin-2 (RhAng2), a competitive antagonist of angiopoeitin-1. Similarly, RhAng2 500 ng/mL treatment promoted MCF7, BT474 and MDA-MB-361 cell proliferations only in endothelial niche, not in mesenchymal niche or in empty plate (tumor cell alone) (Supplementary Fig. 3A, B and C).

### Estrogen depletion negates ER+ tumor cell dormancy in BM endothelial niche via increased angiopoietin-2 signaling

To test the effect of estrogen deficiency on ER+ breast tumor dormancy in BM niche, the growth rates and ERK/p38 activity ratio of breast tumor cells in E_2_-supplemented and unsupplemented niches were compared (Fig. 3A). Three ER+ breast cancer cell lines (MCF7, BT474 and MDA-MB-361) and one ER- cell line (MDA-MB-231) were used. MCF7 represents luminal A (ER+, PR+, HER2-), which is totally dependent on estrogen to grow in culture. BT474 represents luminal B (mild ER+, PR+, strong HER2+), MDA-MB-361 also represents luminal B (ER+, PR+, strong HER2+), both of which are amenable to antiestrogen hormone therapy. MDA-MB-231 represents basal-like subtype (ER&, PR&, HER2&) and is independent of estrogen for growth. When cultured alone, ER+ tumor cells grew significantly slower in absence of E_2_ than in presence of E2 (Fig. 3B), implying that estrogen promotes their growths. Similar growth trends were noted in mesenchymal niche too (Fig. 3B). In endothelial niche, contrastingly, ER+ tumor cells grew significantly faster in absence of E_2_ than in presence of E_2_ (Fig. 3B). Such reverse growth trends were not shown in experiments with ER- tumor cells (Fig. 3B). We also tested the effect of 4-hydroxytamoxifen (4-OHT), an active metabolite of tamoxifen (Mandlekar & Kong 2001), which can effectively suppress ER signaling and ER+ cell growth (Supplementary Fig. 4A and B). Treatment of 4-OHT stimulated MCF7, BT474 and MDA-MB-361 cell growth in endothelial niches, contrary to those cultured alone (Supplementary Fig. 4C and D).

**Figure 4:**
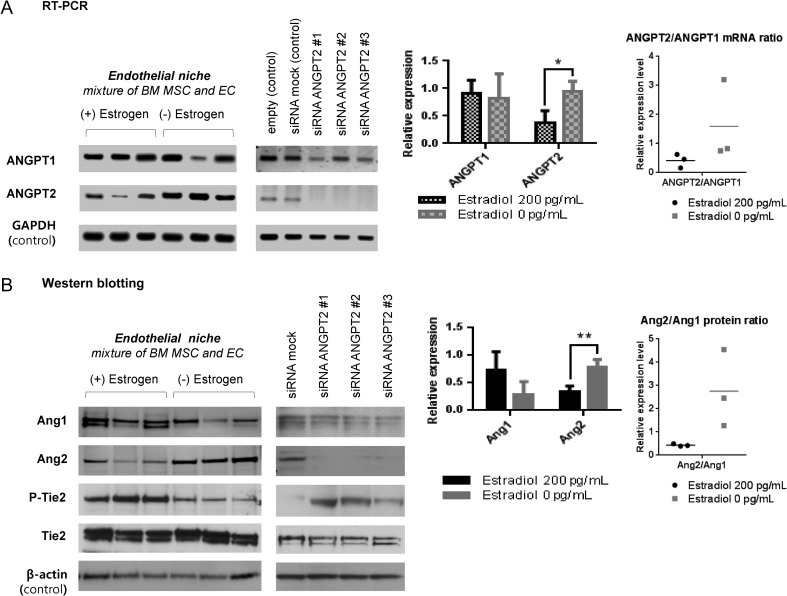
Effect of estrogen depletion on niche endothelial cell angiopoietin-1 and -2 expressions. (A abd B) BM MSC and EC cells were co-cultured with or without estradiol supplementation (200 pg/mL) for 3 days (RT-PCR) or 5 days (Western blotting). Cells were harvested and their expressions of angiopoietin-1, 2 mRNAs (*ANGPT1, 2*) and proteins (Ang1, 2) were compared by RT PCR (A) and Western blot (B). Specific knockdown of *ANGPT2* was performed by transfecting siRNA *ANGPT2* #1, #2, #3 in 6 h after tumor cell seeding. See ‘Materials and methods’ section for siRNA information. Data in graph (left) are presented as mean blot intensity relative to GAPDH (RT-PCR) or &-actin (Western blotting). Data in dot graph (right) are presented as Ang2 blot intensity relative to Ang1. All blots were quantified using ImageJ software (IJ 1.46r). (Three sample sets per group. Error bar: s.d. *P < 0.05.)

We analyzed ERK/p38 activity ratio in ER+ MCF7 cells cultured alone, in mesenchymal niche or endothelial niche (Fig. 3C). Tumor cells cultured alone or in mesenchymal niche showed decreases in awaken population upon estrogen depletion (ERK/p38 activity ratio-high, 89.3 to 20.2% and 43.3 to 17.8%, respectively, Fig. 3C). Meanwhile, tumor cells cultured in endothelial niche exhibited increase in awaken subpopulation upon estrogen depletion (ERK/p38 activity ratio-high; 16.7 to 33.6%, Fig. 3C), suggesting disruption of niche-induced tumor cell dormancy.

The results described above suggest that upon estrogen depletion, some tumor dormancy-related factors in endothelial niche changes. We focused on angiopoietin-1 and 2 specifically. In ER+ breast tumor cells, estrogen downregulates angiopoeitin-1 mRNA in an ER-dependent manner (Harfouche *et al*. 2011). Such regulations may also be active in endothelial cells too. Since the paradoxical growth promoting effect of estrogen depletion was seen only in endothelial niche, we analyzed the expressions of angiopoietin-1, 2 mRNAs and proteins in endothelial niche cells, in E_2_-supplemented and unsupplemented (depleted) conditions. In this experiment, tumor cells were not co-cultured. Angiopoietin-1 levels, as both mRNA and protein forms, were not significantly different in E_2_-supplemented and depleted endothelial niches (Fig. 4A and B). In contrast, angiopoietin-2 mRNA was expressed significantly higher in estrogen-depleted niche than in supplemented niche (Fig. 4A). Protein expression levels exhibited similar trends as well (Fig. 4B). To note, the relative ratio of angiopoietin-2 to angiopoetin-1 level also changed significantly (Fig. 4A and B).

**Figure 5:**
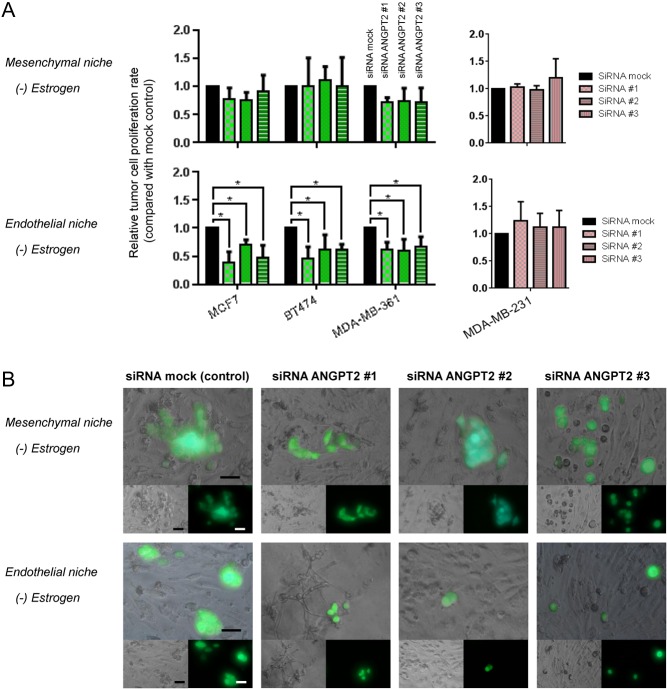
siRNA knockdown of *ANGPT2* suppresses tumor cell proliferation in estrogen-deficient bone marrow endothelial niche. (A and B) GFP-expressing ER+ tumor cell lines were cultured in niches for 3 days with or without estradiol supplementation (200 ng/mL). Specific knockdown of *ANGPT2* mRNA in niche was performed by transfecting siRNA #1, #2 or #3 in 6 h after tumor cell seeding. (A) Relative tumor cell proliferation rates in niches. Cell proliferation rates for 3 days following siRNA transfection were analyzed using fluorescence intensity reader. Data are presented as mean signaling intensity change from day 0 to day 3 relative to siRNA mock control (three sample sets per group; error bar: ±s.d. *P < 0.05, by Student’s *t*-test). (B) Representative images of mesenchymal niches and endothelial niches treated with siRNAs. Images were captured by fluorescence microscope after 3 days of co-culture (Upper: merged; left lower: bright-field; right lower: fluorescence (green). Scale bar = 50 &m). A full colour version of this figure is available at http://dx.doi.org/ 10.1530/ERC-16-0086.

Increased angiopoietin-2 in estrogen-depleted endothelial niche may have shifted tumor cell behavior from dormancy to awakening. This hypothesis was examined by specific knockdown of angiopoietin-2 on niches. Three short-inhibiting RNAs (siRNAs) binding to different angiopoietin-2 mRNA (*ANGPT2*) sequence (siRNA *ANGPT2* #1, #2, #3) significantly attenuated its expressions in endothelial niche (Fig. 4A). Then, tumor cell proliferation rates upon *ANGPT2* knockdown in niche were examined. After tumor cells seeded on to estrogen-deficient endothelial niche and mesenchymal niche, siRNAs were treated. After 72 h, all three ER+ tumor cells in estrogen-deficient endothelial niche did not proliferate when *ANGPT2* was knocked down (Fig. 5A and B). In contrast, *ANGPT2* knockdown had no significant effect on ER+ tumor cell proliferations in estrogen-deficient mesenchymal niche (Fig. 5A and B).

### Angiopoietin-2 promotes ER+ tumor cell survival under estrogen depletion via integrin &1

**Figure 6:**
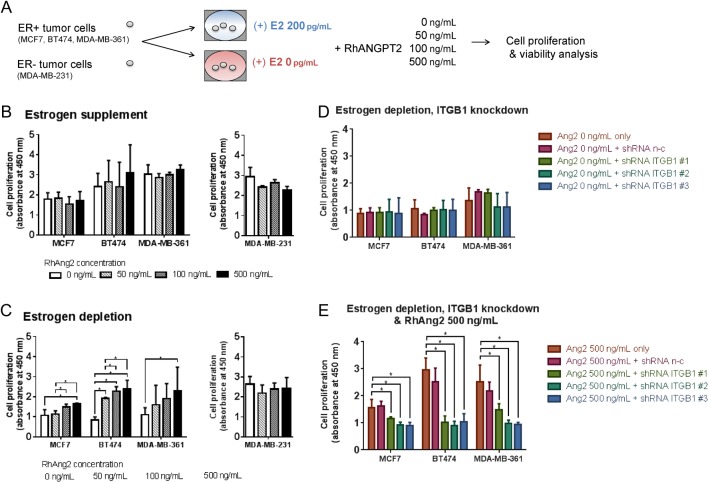
Exogenous angiopoietin-2 promotes ER+ tumor cell survival under estrogen deficiency via cell surface integrin β1. (A) Experimental scheme. ER+ breast tumor cell lines MCF7, BT474, MDA-MB-361 and ER- cell line MDA-MB-231 were cultured in 96-well microplates using estradiol-supplemented (200 pg/mL) or un-supplemented (depleted) media. Recombinant human angiopoietin-2 (RhAng2, 0 ng/mL, 50 ng/mL, 100 ng/mL, 500 ng/mL) were treated 1 h after tumor cell seeding. After 48 h, tumor cell proliferation/viability of each condition was assessed using cell counting kit-8 (Dojindo Molecular Technologies, Rockville, MD, USA). (B, C and D) Tumor cell proliferation/viability in presence of RhAng2. (B) Tumor cells cultured in estrogensupplemented media. (C) Tumor cells cultured in estrogen-depleted media. (D) Integrin β1 (ITGB1) knockdown tumor cells and untreated cells cultured in estrogen-depleted media. Integrin β1 knockdown was performed using short-hairpin RNAs (shRNAs), as described in Supplementary Fig. 6 and previously (Choi *et al*. 2012). Data presented are mean values of light absorbance rate at 450 nm, representing proliferative activity of each well; error bar: ±s.d. *P < 0.05. A full colour version of this figure is available at http://dx.doi.org/10.1530/ERC-16-0086.

As described above, RhAng2 treatment (500 ng/mL) increased ER+ tumor cell proliferations in otherwise growth-suppressing E_2_-supplemented endothelial niches (Supplementary Fig. 3A, B and C). RhAng2 had no effect on their proliferations in E_2_-supplemented mesenchymal niche (Supplementary Fig. 3A, B and C). However, in estrogen-depleted mesenchymal niches, RhAng2 increased tumor cell proliferations, although not always in significant degree and in dose-dependent manner (Supplementary Fig. 2). Since there is no Ang2-responsive ECs in mesenchymal niche, we hypothesized that angiopoietin-2 may act on tumor cells directly. Interestingly, Imanishi and coworkers (2007, 2011) reported that angiopoeitin-2 acts directly on ER+ tumor cells via ITGB1 and promoted their initial survival and metastatic growth at lung. Encouraged by the previous reports, we evaluated the effect of RhAng2 on ER+ tumor cell proliferation under estrogen deficiency. Tumor cells were cultured for 3 days in E_2_-supplemented or unsupplemented growth media containing varying concentrations of RhAng2 (0, 50, 100, 500 ng/mL, Fig. 6A). In presence of E_2_, RhAng2 had no effect on ER+ and ER- tumor cell proliferations (Fig. 6B). In absence of E_2_, however, RhAng2 promoted ER+ tumor cell proliferations in dose-dependent manner (Fig. 6C). ER- tumor cell proliferation was not affected by RhAng2 (Fig. 6C). To find-out whether this was dependent on tumor cell ITGB1, we performed ITGB1 knockdown using shRNAs (Supplementary Fig. 6). ITGB1 knockdown itself had no effect on tumor cell growth under estrogen deficiency (Supplementary Fig. 6D). In contrast, ITGB1 knockdown completely negated RhAng2’s proliferative effect under estrogen depletion (Fig. 6E).

### Angiopoietin-1, 2 expressions correlate with ER+ breast cancer metastatic recurrences following antiestrogen endocrine therapy

**Figure 7:**
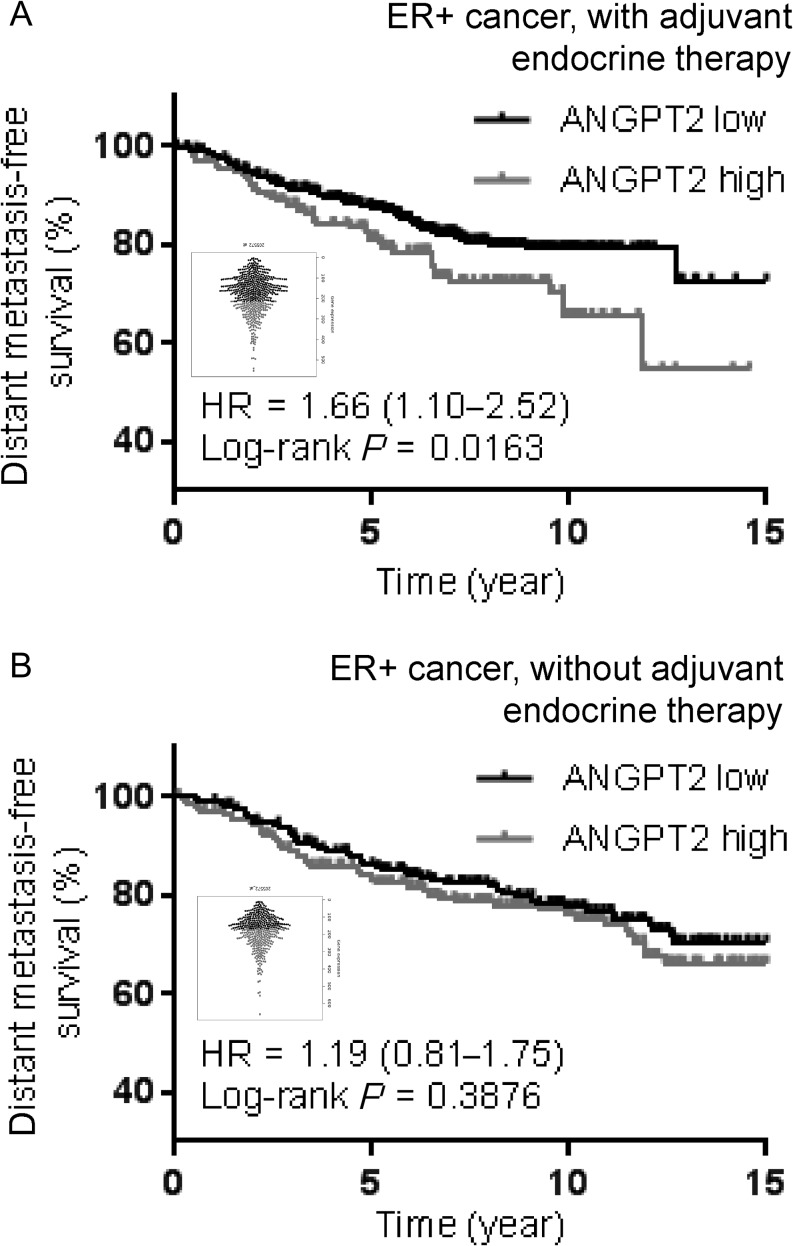
Distant metastasis-free survival of ER+ breast cancer patients, stratified by *ANGPT2* expression. Kaplan–Meier analysis comparing distant metastasis-free survival of ER+ breast cancer patients following endocrine therapy (*n* = 561, A) and those without endocrine therapy (*n* = 435, B), distinguished by low versus high expressions of *ANGPT2*. Gene expression data obtained from the open-source KM Plotter (Szasz *et al*. 2016). Beeswarm graph plots of each RNA prove distribution. *ANGPT2*, angiopoietin-2 mRNA; ER, estrogen receptor.

Angiopoietin-2 overexpression in breast cancer is known to correlate with poor patient survival (Sfiligoi *et al*. 2003). According to our experimental data, its expression would be particularly associated with metastatic recurrence of ER+ subtype after adjuvant antiestrogen therapy. To test the hypothesis, we used a published dataset and a web-based program ‘KM plotter’ (http://kmplot.com) that contain both gene expression and human patient survival data (Szasz *et al*. 2016). Patients were first categorized into those who underwent adjuvant endocrine therapy (including estrogen receptor blocker tamoxifen and aromatase inhibitor, *n *= 561) or those who did not undergo endocrine therapy (*n* = 435). Then, the patients were further stratified into two groups based on angiopoietin-2 mRNA (ANGPT2) levels in tumor tissue. For those who underwent antiestrogen endocrine therapy, increased *ANGPT2* level was associated with a significantly decreased probability of distant metastasis-free survival (DMFS) (Fig. 7A). For those without endocrine therapy, contrastingly, *ANGPT2* level was not associated with the probability of DMFS (Fig. 7B). We did not perform a multivariate analysis incorporating known prognostic indicators such as tumor size, grade and number of axillary lymph nodes involved.

## Discussion

The objective of this study was to investigate the effects of estrogen deficiency on BM microenvironment and dormancy of residing ER+ breast tumor cells. We found that prolonged estrogen depletion turned the BM niche in to pro-tumorigenic state, mainly by upregulating angiopoietin-2. Angiopoietin-2 ameliorated the dormancy of ER+ tumor cells in BM endothelial niche and promoted their survival under estrogen deficiency via integrin &1 (ITGB1).

Physiologically, angiopoietin-2 destabilizes microvasculature and promotes angiogenesis, via interfering cell surface Tie2 receptor binding to angiopoeitin-1 or activating integrin &1 (Augustin *et al*. 2009, Hakanpaa *et al*. 2015). Its overexpression has been noted in many types of tumors and linked to poor prognosis (Eroglu *et al*. 2013). In ovarian cancer, angiopoietin promotes intraperitoneal tumor growth by accumulating cancer-promoting fibroblasts and enhancing tumor angiogenesis (Brunckhorst *et al*. 2014). In pancreatic cancer, angiopoietin-2 drives lymphatic metastasis (Schulz *et al*. 2011). In breast cancer, it impairs the blood–brain barrier and support tumor cell colonization in brain (Avraham *et al*. 2014). In our study, angiopoeitin-2 blocked endothelial Tie2 receptor activity, promoting ER+ tumor cell growth in otherwise growth-suppressing endothelial niche. Thromospondin-1 (TSP-1) is the endothelium-derived growth inhibitory molecule acting on dormant breast tumor cells (Ghajar *et al*. 2013). Its level in endothelial niche correlated with the Tie2 receptor activity, suggesting it is working in our model too. However, this is in contrast to a previous report that inhibition of Tie2 signaling induces endothelial TSP-1 overexpression (Niu *et al*. 2004). Our interpretation is that during angiogenesis, endothelial Tie2 activation promotes angiogenesis, lowering TSP-1 production; after vessels formed, endothelial Tie2 activation promotes vessel maturation (Hawighorst *et al*. 2002), in which TSP-1 production is increased.

Our results underscore the direct action of angiopoietin-2 on ER+ tumor cells too. Unlike Tie2 receptor, which is expressed exclusively in ECs, ITGB1 is expressed in most epithelial tumor cells (Guo & Giancotti 2004). We showed that angiopoeitin-2 supported estrogen-independent tumor cell growth via ITGB1. Interestingly, it has been known that the activation of ITGB1 turns dormant tumor cells into awakening state (White *et al*. 2004). In addition, angiopoietin-2 can stimulate tumor cell metastasis to lymph nodes and lung, via activating tumor cell ITGB1 (Imanishi *et al*. 2007). When those findings combined, it is likely that inactivation of angiopoietin-2 signaling would effectively prevent the metastatic recurrence of breast cancer, particularly of ER+ subtype.

Our study indicates that estrogen regulates angiopoietin-2 expression in BM microenvironment. There appears to be a complex relationship between estrogen level and angiopoietin-1, 2 expressions. Glinskii and coworkers (2007) reported that cessation of ovarian hormone significantly reduced angiopoietin-1 expression in meningeal microvasculature. Similarly, Ardelt and coworkers (2005) reported that estradiol (E2) increased angiopoietin-1 expression in cerebral vascular beds, and Bonagura and coworkers (2010) reported that E_2_ increased angiopoietin-1 expression in endometrial glandular epithelial cells. In contrast, other reports showed that estrogen administration reduced angiopoietin-1 levels in placenta (Albrecht *et al*. 2008), in ER+ breast tumor cells (Harfouche *et al*. 2011) and in some nonreproductive organs (Ye *et al*. 2002). Furthermore, E_2_ treatment transiently increased angiopoietin-2 expression in ER+ T47D and ZR75.1 cells, while estrogen depletion also increased angiopoeitin-2 expression in these cells slowly but gradually (Sfiligoi *et al*. 2003). These discrepancies may reflect differences in experimental conditions and tissue-specific regulatory mechanisms. Nevertheless, all studies suggest that sudden changes in estrogen levels do affect angiopoietin-2 expressions. Therefore, it will be valuable to compare systemic and local angiopoietin-2 levels in pre- and postmenopausal women of breast cancer. Systemic angiopoietin-2 levels may be used as an alarm of disseminated tumor cell awakening too.

The *in vitro* culture system described here further limits the results of our study. Also, it should be borne in mind that in clinic, macroscopic metastases of ER+ breast cancer may present at a time that there is no apparent change in estrogen levels (Brackstone *et al*. 2007). Moreover, the experimental condition of rapid estrogen withdrawal is not necessarily same as the long-term low estrogen environment of adjuvant endocrine therapy setting. Therefore, these findings are purely experimental. However, we believe that the experimental design and results are consistent with existing *in vivo* models and results from human patient studies. We would like to state that any conditional overexpression of angiopoeitin-2, regardless of estrogen level, can provoke metastatic reactivation of solitarily disseminated tumor cells in estrogen-depleted bone microenvironment. Further, preclinical studies using angiopoietin-2 inhibitors in the premetastatic setting could add significance on our findings.

Treatment strategies for primary breast cancer have been successful. However, for metastatic disease, currently available therapeutic tools are still considered inadequate. ER+ breast cancer is of particular concern because its metastasis often precedes a period of dormancy. Dormant ER+ tumor cells in BM niche are thought to be the seed of metastasis. They are rare and difficult to identify and monitor using currently available methods, and are protected by the microenvironment and their own unique state from conventional cytotoxic chemotherapies. Conversely, dormant tumor cells are not harmful until they become activated and proliferate to form a tumor. Therefore, presence of dormant tumor cells also indicates an opportunity to control metastatic disease even before becoming apparent. Based on current findings, further studies are mandatory to verify the effect of angiopoietin-2 signaling manipulation on late recurrence prevention in women with breast cancer, particularly those under risk of estrogen deficiency.

## Supplementary data

This is linked to the online version of the paper at http://dx.doi.org/10.1530/ERC-16-0086.﻿

## Declaration of interest

The authors declare that there is no conflict of interest that could be perceived as prejudicing the impartiality of the research reported.

## Funding

This study was supported by the Mid-Career Researcher Program through a National Research Foundation of Korea grant (No. 2016R1A2B4011115; CNH) and by a faculty research grant from the Yonsei University College of Medicine (6-2015-0040; CNH). This work was also supported by the Brain Korea 21 PLUS Project for Medical Science, Yonsei University.

## Author contribution statement

H H contributed to experimental conception, design, data acquisition, analysis and interpretation, manuscript drafting and revision. B K, J L, S K and J K contributed to experimental design, data acquisition and interpretation. N C contributed to experimental conception, data interpretation and manuscript revision.
